# Supplementary figures and images for: A convenient diagnostic tool for discriminating adult-onset glutamic acid decarboxylase antibody-positive autoimmune diabetes from type 2 diabetes: a retrospective study
Source: PeerJ. 2020 Feb 14;8:e8610. doi: 10.7717/peerj.8610 (PMC7025710; doi:10.7717/peerj.8610)

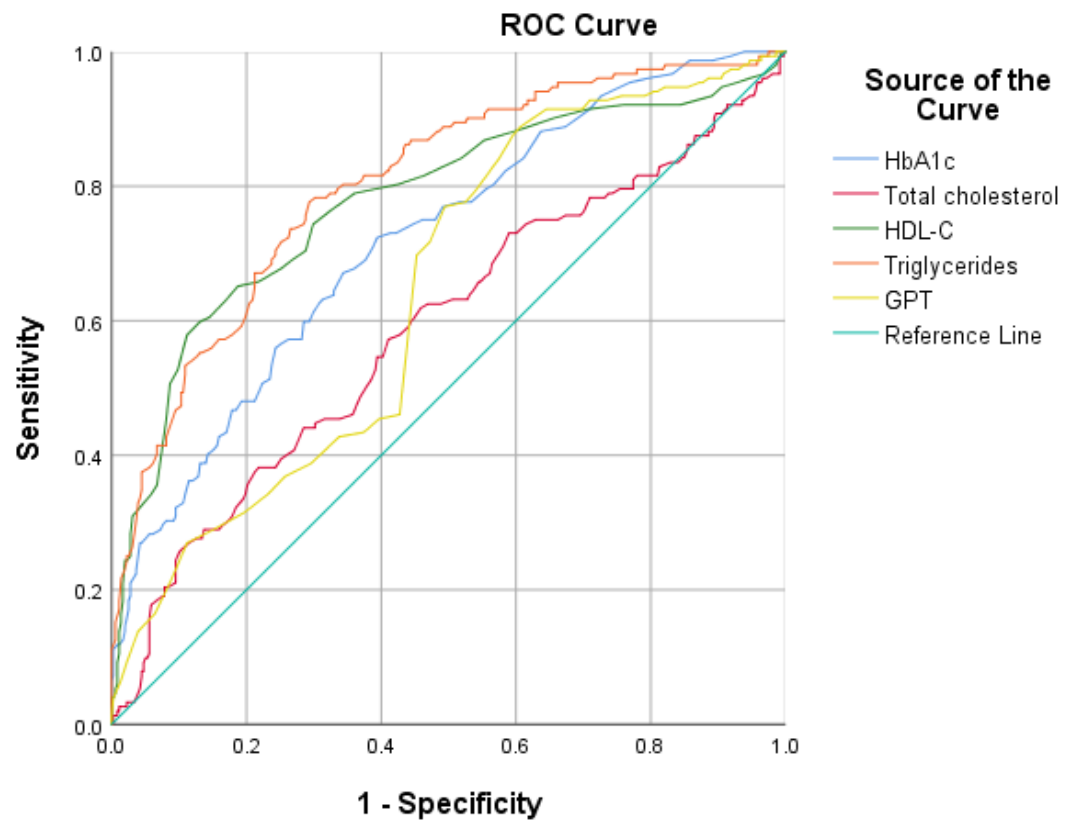

Supplement: Figure S1 — Abbreviations: GADA, Glutamic acid decarboxylase antibody; T2DM, type 2 diabetes mellitus; HDL-C, high-density lipoprotein cholesterol; HbA1c, hemoglobin A1c; GPT, glutamic pyruvic transaminase. [file peerj-08-8610-s001.pdf]
